# Pre-treatment with sumatriptan for cilostazol induced headache in healthy volunteers

**DOI:** 10.1186/s10194-018-0890-y

**Published:** 2018-08-17

**Authors:** Katrine Falkenberg, Jes Olesen

**Affiliations:** 1 Danish Headache Center and Department of Neurology, University of Copenhagen, Rigshospitalet Glostrup, Copenhagen, Denmark; 2 Danish Headache Center, Department of Neurology N39, Rigshospitalet Glostrup, University of Copenhagen, Valdemar Hansens vej 5, DK-2600 Glostrup, Denmark

**Keywords:** Headache, Migraine, Pain, Phosphodiesterase type 3, Human migraine model

## Abstract

**Background:**

Previous studies indicate that sumatriptan is not effective when second messenger levels are high as after cilostazol provocation. Therefore, we have conducted the present study, where sumatriptan is administrated as pretreatment before cAMP increases due to cilostazol intake. Our hypothesis was that pretreatment with sumatriptan would have a significant effect against cilostazol induced headache in healthy volunteers.

**Methods:**

In a double-blind, randomized, crossover design, 30 healthy volunteers of both sexes received cilostazol 200 mg on two separate days, each day preceded by oral sumatriptan (2 × 50 mg) or placebo. Headache response and accompanying symptoms were registered in a questionnaire by the participants themselves.

**Results:**

Cilostazol induced a mild to moderate headache in all but 3 participants (Range 0–7 on Numerical Rating Scale). There was no significant difference in headache score 2 h (*p* = 0.67) or 4 h (*p* = 0.1) after treatment between the 2 days. Median peak headache score was 1.5 (range 0–5) on the sumatriptan day and 2 (range 0–7) on the placebo day (*p =* 0.26).

**Conclusion:**

Pre-treatment with sumatriptan prevents cilostazol induced headache from developing. However, the placebo group did not develop enough headache to get statistical significant results. The cilostazol pre-treatment model is valuable for experimental headache research and perhaps for testing drugs with another mechanism of action.

**Trial registration:**

ClinicalTrials.gov Identifier: NCT03156920.

**Electronic supplementary material:**

The online version of this article (10.1186/s10194-018-0890-y) contains supplementary material, which is available to authorized users.

## Background

In recent years, the authors made a dedicated attempt to develop a pragmatic model for testing new anti-migraine drugs. The idea behind the model is to have participants come to the hospital and to be able to induce migraine attacks and treat them. That would enable the drug industry to test their new anti-migraine drug under controlled conditions, inexpensively and in a short period of time. We have tried to develop the model in both healthy volunteers and in patients with migraine without aura (MO) using two different headache inducing substances; Isosorbide-5-mononitrate (5-ISMN, a long lasting NO-donor) and cilostazol (a phosphodiesterase 3- inhibitor).

NO leads to an increase in cGMP in the cell and cilostazol increases cAMP [1, 2]. To validate the model, we tested the effect of sumatriptan on the induced headache. Sumatriptan had no effect on 5-ISMN induced headache [3]. When treating cilostazol induced headache in healthy volunteers with sumatriptan, we saw a trend towards an effect [4] and the trend was stronger when the same study was conducted in MO patients [5]. However, all three studies indicate that sumatriptan is not effective when second messenger levels (cGMP and cAMP), are increased in the system. Therefore, we have conducted the present study, where sumatriptan is administrated as pretreatment before cAMP increases due to cilostazol intake. Our hypothesis was that pretreatment with sumatriptan would have a significant effect against cilostazol induced headache in healthy volunteers.

## Methods

A similar method has been described previously in three studies by the authors [3–5].

### Participants

Thirty healthy volunteers (15F/15M) with no history of migraine were included. Inclusion criteria were: healthy subjects of both sexes aged 18–60 years and weighting 45–90 kg.

Exclusion criteria were: any type of headache (except episodic tension-type headache < 1 day per week), serious somatic or psychiatric disease, pregnancy, and intake of daily medication (except oral contraceptives). The participants were informed that cilostazol might induce headache or migraine in some individuals.

### Design

We conducted a double-blinded, randomized, balanced, placebo-controlled, cross-over study. The participants received pre-treatment with 50 mg of sumatriptan or placebo 1.5 h prior to oral cilostazol of 200 mg. Together with cilostazol intake, the participants took an additional 50 mg of sumatriptan or placebo. Tmax for both sumatriptan and cilostazol is about 2 h and we thus reach maximum concentration of sumatriptan and cilostazol at the same time. The reason why we also gave sumatriptan 1.5 h prior to cilostazol was to have a high dose of sumatriptan at time of cilostazol intake. Cilostazol dose (200 mg) was chosen based on previous headache-studies.

The central pharmacy of the Capital Region of Copenhagen performed the randomization of the experimental drug in a balanced fashion. The randomization code did not leave the hospital during the study and was not available to the investigators until after termination of the study. We did not break the code until data management took place.

### Standard protocol approvals

All participants gave written, informed consent to participate in the study. The study was approved by the Ethics Committee of Copenhagen (H-15011960) and the Danish Data Protection Agency. The study is registered on clinicaltrials.gov (NCT03156920) and was conducted according to the Helsinki II declaration of 1964, as revised in 2008.

All participants were enrolled via the website forsøgsperson.dk [6].

### Study procedure

The participants had to be headache free 48 h prior to the study and not to have taken any type of painkillers 12 h before beginning of the study. A pregnancy test was taken at the beginning of each study day on all fertile female participants. All participants had two separate study days at least five days apart. They arrived non-fasting at the clinic between 8:00 a.m. and 12:00 p.m. Full medical history, physical examination, electrocardiography (ECG), vital signs and baseline headache were collected at arrival. All participants received the treatment (placebo or sumatriptan) and waited for 1.5 h before they received cilostazol 200 mg orally together with an additional pill of treatment (the same treatment as the first one). Hereafter the participants were discharged from hospital. In case of severe headache not responding to the experimental treatment, the participants were allowed rescue with nonsteroidal anti-inflammatory drugs (NSAIDs) and paracetamol, but not before two hours after cilostazol. During the study, an emergency phone was always open where participants could call if they experienced severe headaches or discomfort.

### Headache parameters

Headache parameters and accompanying symptoms were recorded by the investigator at baseline on a headache questionnaire. Afterward headache intensity, characteristics (unilateral/bilateral, quality and aggravation by physical activity), accompanying symptoms (nausea/vomiting, phono- and photophobia) and side effects were scored on a self-administered questionnaire. The participants had to fill out the questionnaire every 30 min the first 6 h after cilostazol and thereafter every hour until 12 h after cilostazol. The intensity was scored on a Numerical Rating Scale (NRS) from 0 to 10, 1 representing a very mild headache (including feeling of pressing or pulsation), 5 a headache of medium severity and 10 the worst possible headache (10). Missing data were filled in using last observation carried forward.

The following criterion was used for a migraine-like attack induced 0–12 h after administration of cilostazol:

Headache fulfilling criteria C and D for migraine without aura according to the IHS criteria [7].

C. Headache has at least two of the following characteristics:

- Unilateral location
- Pulsating quality
- Moderate or severe pain intensity (moderate to severe pain intensity is considered ≥4 on NRS)
- Aggravation by cough or causing avoidance of routine physical activity

D. During headache at least one of the following:

- Nausea and/or vomiting
- Photophobia and phonophobia.

### Statistical analysis

Calculation of sample size was based on the detection of a difference in headache intensity between two experimental days, at 5% significance with 90% power. We estimated that placebo had an effect on 20% and sumatriptan had 60% effect. Standard deviation was estimated based on previous data. The correlation between the 2 days was estimated conservatively at 0.5. We also assumed no carry-over effect. We calculated that at least 18 participants should complete both experimental days. Due to uncertainty regarding these assumptions we decided to include 30 participants. The area under the curve (AUC) for headache score was used as a summary measure for analyzing differences between the groups and was calculated according to the trapezium rule (12). Our primary endpoints were (1) difference in pain intensity difference between sumatriptan and placebo 2 hours after the last treatment, and (2) difference in AUC 0-4 h between the two experimental days. Secondary end-points were difference in median peak headache score, difference in median headache intensity between the two treatments at 4 h, AUC 0-2 h after sumatriptan/placebo and accompanying symptoms as nausea, photo- and photophobia.

Headache intensity scores are presented as medians (range). Differences in AUC for headache scores were tested using the Wilcoxon signed rank test. Difference in pain intensity difference between the sumatriptan day and the placebo day were tested using Mann-Whitney test. The incidence of headache and associated symptoms were analyzed as binary categorical data with McNemar’s test. Age and weigh are presented as means. All analyses were performed with SPSS for Windows 11.5 (Chicago, IL, USA), or GraphPad Prism version 7.0. A *p* < 0.05 was considered significant.

## Results

Thirty participants (15F, 15 M) with a mean age of 26.5 (19–58 years) and a mean weight on 71.6 kg (48-93 kg), completed the study. Three participants had a first degree relative with migraine (self-reported).

Cilostazol induced a mild to moderate headache (range 1–7 on NRS) in all but three participants. The headache had some migraine features, especially throbbing character and it was aggravated by physical activity (see Table 1 for clinical characteristics of the headache and associated symptoms). Median peak headache score was 1.5 (range 0–5) on the sumatriptan day and 2 (range 0–7) on the placebo day. Median time to peak headache score was 5 h and 4.5 h respectively. Two subjects in each group experienced a migraine-like attack with a mean time of onset of 8.5 h (7 and 10 h) on the sumatriptan day and 4 h (1 and 7 h) on the placebo day.

**Table 1:** Clinical characteristics (our secondary end-point) of headache and associated symptoms after cilostazol

	Sumatriptan (*n* = 30)	Placebo (*n* = 30)	*P*-value*
Number of participants reported headache	27	27	1.00
(range 1–7 on VRS)			
Median peak headache score (range)	1.5 (0–5)	2 (0–7)	0.26**
No. of participants with			
Throbbing headache	13	15	0.77
Unilateral location	10	8	0.77
Nausea	7	6***	1.00
Aggravation by physical activity	15	17	0.79
Photophobia	7	5	0.72
Phonophobia	4	2	0.61
Rescue medication	0	2	0.48
Migraine-like attack	2	2	1.00

* McNemar’s test

** Wilcoxon signed rank test

*** Two subject vomited

**Fig. 1:**
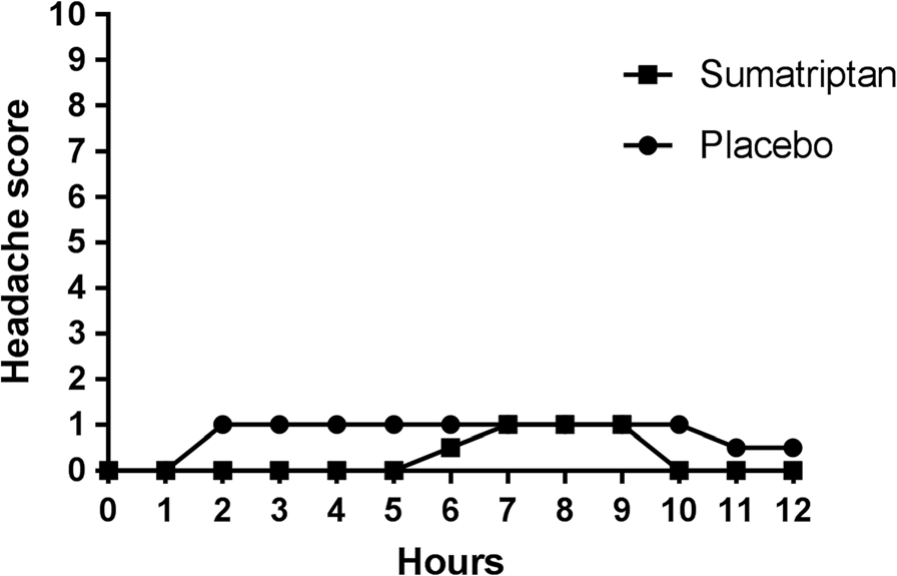
Headache score 0-12 h after cilostazol. Median headache score 0-12 h after cilostazol. Median headache score was 1 on both days and thus we found no difference between the two treatment days

**Fig. 2:**
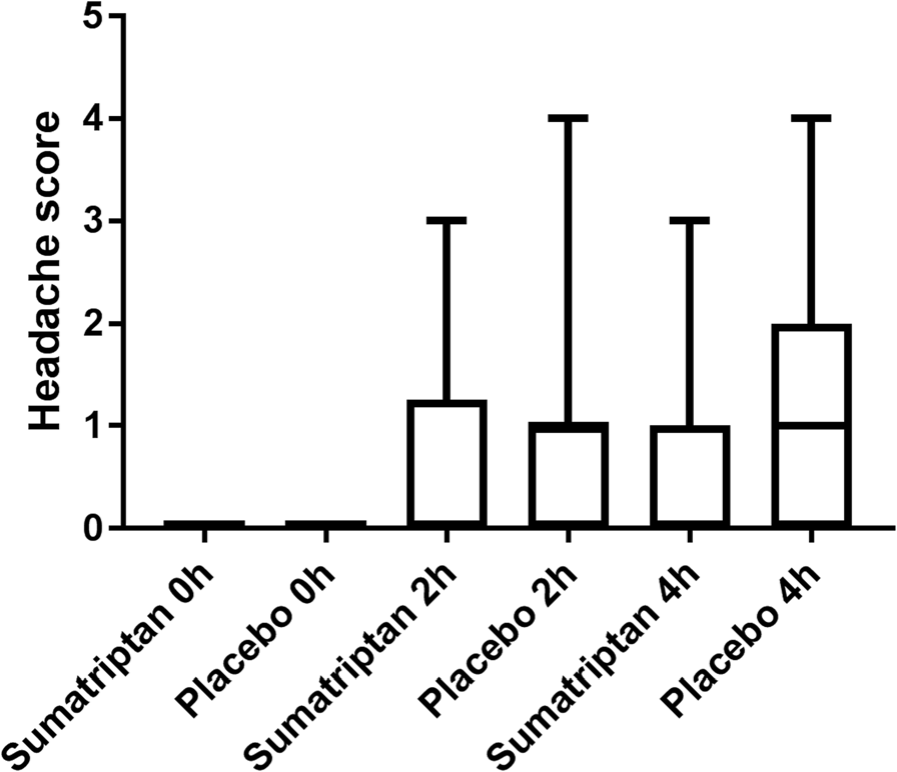
Headache score after treatment. Headache score 0 h, 2 h and 4 h after treatment with placebo and sumatriptan. There was no difference in headache score at any time-point between the two treatment days

There was no significant difference in headache score between the 2 days at 2 h after treatment (*p* = 0.52) and thus our primary end-point was negative. Two hours after treatment intake, median headache score was 0 (range 0–3) for the sumatriptan day and 1 (range 0–4) for the placebo day. At 4 h after treatment intake (our secondary end-point) there was no significant difference either (*p* = 0.06). Median headache score 0-12 h after cilostazol for the two treatment groups is illustrated in Fig. 1 and headache score after treatment is illustrated in Fig. 2.

**Fig. 3:**
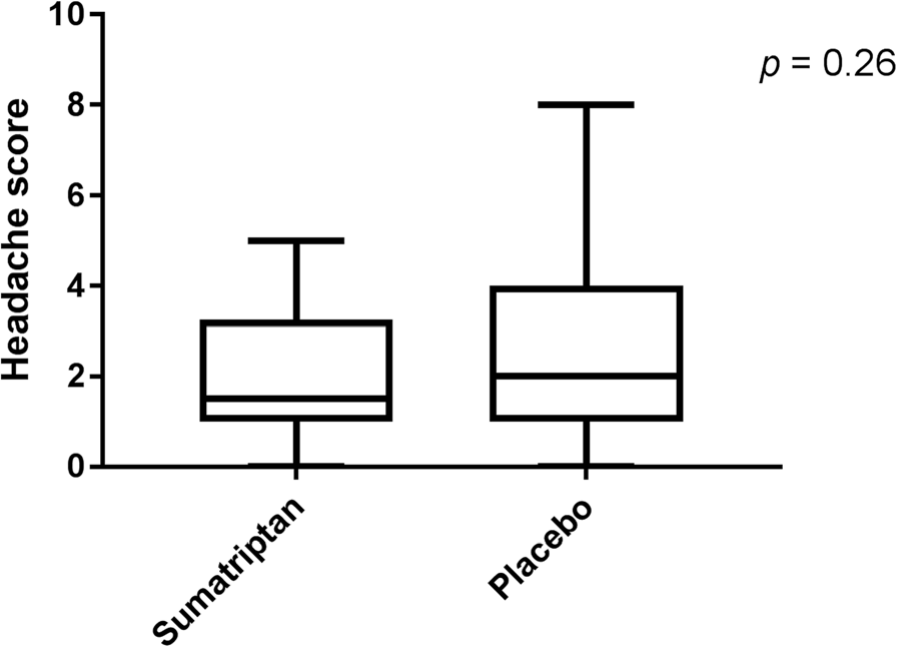
Peak headache score. Difference in median peak headache score on the two treatment days was not statistical significant

Our other primary end point, AUC 0-4 h after treatment did not differ between the 2 days (*p =* 0.57). Nor did we see a difference in our secondary end point, difference in peak headache score (*p =* 0.26) see Fig. 3.

## Discussion

Cilostazol induced a mild to moderate headache in 27/30 participants. The median headache score after sumatriptan was 0 and thus sumatriptan may have prevented the headache from developing. However, our end-points were all statistically negative because the placebo group did not develop much headache either (median headache score at 1) (See Fig. 1).

### Modifying the model from previous studies

In previous studies, we showed a trend towards an effect of sumatriptan on cilostazol induced headache in both healthy volunteers and in MO patients [2, 3]. The effect was, however, not clear and the model needed modification in order to be valid for future drug testing. Cilostazol induces headache by an increase of cAMP which leads to vasodilatation and sensitization of nerve endings. One possible explanation for the lack of effect of sumatriptan in our previous studies could be that sumatriptan cannot exert its effect when cGMP and cAMP have already accumulated in the cell, which happens after provocation with isosorbide-5-mononitrate and cilostazol respectively [1–3].

Cilostazol inhibits phosphodiesterase 3 and thus prevents cAMP break-down. Therefore, in order for cAMP to be accumulated in the cell due to cilostazol, there must be cAMP in the cell beforehand. Sumatriptan inhibits adenylate cyclase leading to a decreased formation of cAMP in the cell. In the present study we tried to modify the previous model by administering sumatriptan as pretreatment and thus impeding the formation of cAMP before cilostazol provocation. We hypothesized that there would be no cAMP to accumulate in the cell and therefore no headache would occur. Our results suggest that sumatriptan pre-treatment may prevent the headache from developing since median headache score remained 0 until 6 h after sumatriptan. At 6 h the effect of sumatriptan has subsided and the headache score increased to 1, like in the placebo group (see Fig. 1). An explanation why our results all came out statistically negative is that we have a considerably lower headache induction in the present study, compared to previous studies (see elaboration below).

### The headache inducing properties of cilostazol in healthy volunteers

It has previously been described that cilostazol induces a headache with migraine characteristics in healthy volunteers and a migraine-like attack in migraine patients [2, 8]. More and more studies are using cilostazol headache models to study the pathophysiology of migraine. Nevertheless, the headache inducing property of cilostazol differs depending on study design.

Our own group has completed 3 such studies: Birk et al. conducted a double-blind randomized placebo-controlled crossover study in which the participant received cilostazol on 1 day and placebo on the other day. The participants were not offered any treatment as part of the study. Cilostazol induced a median peak headache score of 3.5 on NRS.

In a previous study by the present authors, cilostazol induced headache, once established, was treated with sumatriptan and placebo in a double-blind crossover study. Treatment was then given on both study days. Median peak headache score was 2 on the sumatriptan day and 3 on the placebo day. Hence, when the participants receive treatment for the headache (including placebo) we see a milder headache score than if no treatment is offered.

In keeping with that result, the present study where sumatriptan and placebo were given as pretreatment found, median peak headache score of only 1.5 on the sumatriptan day and 2 on the placebo day. When treatment is administered before the headache inducing substance, the placebo effect may thus be even bigger. It is interesting, that the placebo effect seems to be present in experimental headache induction studies to a degree similar to spontaneous migraine attacks.

### Can we use the present model for drug testing?

We suggest that the cilostazol pre-treatment model is valuable for experimental headache research and perhaps for novel drug testing, but the model needs some modifications. It could for an example be interesting to conduct the present study on migraine patients, who are known to develop more headache from cilostazol than healthy volunteers.

## Conclusion

This study shows that pre-treatment with sumatriptan prevents cilostazol induced headache from developing. However, the placebo group did not develop enough headache to get statistically significant results. We also show by comparison to previous studies that the placebo effect on experimental headache is considerable and should be taken into consideration in future power calculations.

## Additional file

Additional file 1: Datasheet pre-treatment. (XLSX 18 kb)

## Abbreviations

5-ISMN: Isosorbide-5-mononitrate
AUC: Area under the curve
cAMP: Cyclic adenylate monophosphate
cGMP: Cyclic guanosine monophosphate
ECG: Electrocardiography
IHS: International Headache Society
MO: Migraine without aura
NRS: Numerical rating scale
NSAIDs: Nonsteroidal anti-inflammatory drugs
PDE3: Phosphodiesterase 3
PID: Pain intensity difference
